# How to avoid unintended valgus alignment in distal femoral derotational osteotomy for treatment of femoral torsional malalignment - a concept study

**DOI:** 10.1186/s12891-017-1904-7

**Published:** 2017-12-29

**Authors:** Florian B. Imhoff, Bastian Scheiderer, Philip Zakko, Elifho Obopilwe, Franz Liska, Andreas B. Imhoff, Augustus D. Mazzocca, Robert A. Arciero, Knut Beitzel

**Affiliations:** 1 Department of Orthopaedic Sports Medicine, Technical University, Munich, Germany; 2 Uconn Health, Department of Orthopaedic Surgery, Farmington, CT USA

**Keywords:** Distal femoral derotational osteotomy, Patellofemoral instability, Valgus-varus alignment, Torsion correction, Mechanical anatomical axis

## Abstract

**Background:**

Defining the optimal cutting plane for derotational osteotomy at the distal femur for correction of torsion in cases of patellofemoral instability is still challenging. This preliminary study investigates changes of frontal alignment by a simplified trigonometrical model and demonstrates a surgical guidance technique with the use of femur cadavers. The hypothesis was that regardless of midshaft bowing, a cutting plane perpendicular to the virtual anatomic shaft axis avoids unintended valgus malalignment due to derotation.

**Methods:**

A novel mathematical model, called the Pillar-Crane-Model, was developed to forecast changes on frontal alignment of the femur when a perpendicular cutting plane to the virtual anatomical shaft was chosen. As proof of concept, eight different torsion angles were assessed on two human cadaver femora (left and right). A single cut distal femoral osteotomy perpendicular to the virtual anatomical shaft was performed. Frontal plane alignment (mLDFA, aLDFA, AMA) was radiographically analyzed before and after rotation by 0°, 10°, 20°, and 30°. Measurements were compared to the model.

**Results:**

The trigonometrical equation from the Pillar-Crane-Model provides mathematical proof that slight changes into varus occur, seen by an increase in AMA and mLDFA, when the cutting plane is perpendicular to the virtual anatomical shaft axis. A table with standardized values is provided. Exemplarily, the specimens showed a mean increase of AMA from 4.8° to 6.3° and mLDFA from 85.2° to 86.7 after derotation by 30°. Throughout the derotation procedure, aLDFA remained at 80.4° ± 0.4°SD.

**Conclusions:**

With the use of this model for surgical guidance and anatomic reference, unintended valgus changes on frontal malalignment can be avoided. When the cutting plane is considered to be perpendicular to the virtual anatomical shaft from a frontal and lateral view, a slight increase of mLDFA results when a derotational osteotomy of the distal femur is performed.

## Background

Increased femoral antetorsion is one important risk factor for patellofemoral instability and anterior knee pain syndrome in teenagers and young adults [1–5]. Derotational osteotomy, a procedure involving external rotation of the distal femur, is a reliable option for correction of torsional pathologies [6–9]. Suggestions for positioning and orientation of the osteotomy to the shaft vary widely in the literature, and no consistent reference for orientation of the cutting plane can be found [3, 10]. A recent study showed increased valgus producing effects when distal derotational osteotomies were performed with regards to the anatomical shaft axis and its bowing in a computed model [11]. Increased valgus alignment could lead to increased lateral facet pressure and increased medial retinaculum strain [12–14]. In the clinical experience and with regards to previous publications, derotational osteotomies may result in unplanned frontal malalignment; thus, unintended valgus producing effects should be avoided strictly [11, 15, 16].

Femoral mechanical and anatomical axes differ and their angulation and relation may be altered due to rotational osteotomies, as Paley described [17]. However, Strecker et al. described that a cutting plane perpendicular to the mechanical axis will not have any impact on the mechanical axis when rotation is performed [3]. Unfortunately, verification of the mechanical axis in a clinical setup and related anatomic reference may not be reliable for an unexperienced surgeon. To overcome this, the Pillar-Crane-Model is introduced, which describes a virtual anatomical axis whereby a perpendicular rotation axis to the virtual anatomical axis provides an ideal cutting plane.

The hypothesis was that regardless of midshaft bowing, this model helps avoid unintended valgus malalignment on the frontal plane while simultaneously slightly increasing varus of the mechanical axis.

The purpose of this study is to investigate changes on frontal alignment at different torsion angles using trigonometrical calculations, and to exemplarily show on femur cadavers that a cutting plane perpendicular to the virtual anatomical shaft will lead to a slight varus change on the frontal plane alignment.

## Methods

### Pillar-crane-model

**Fig. 1:**
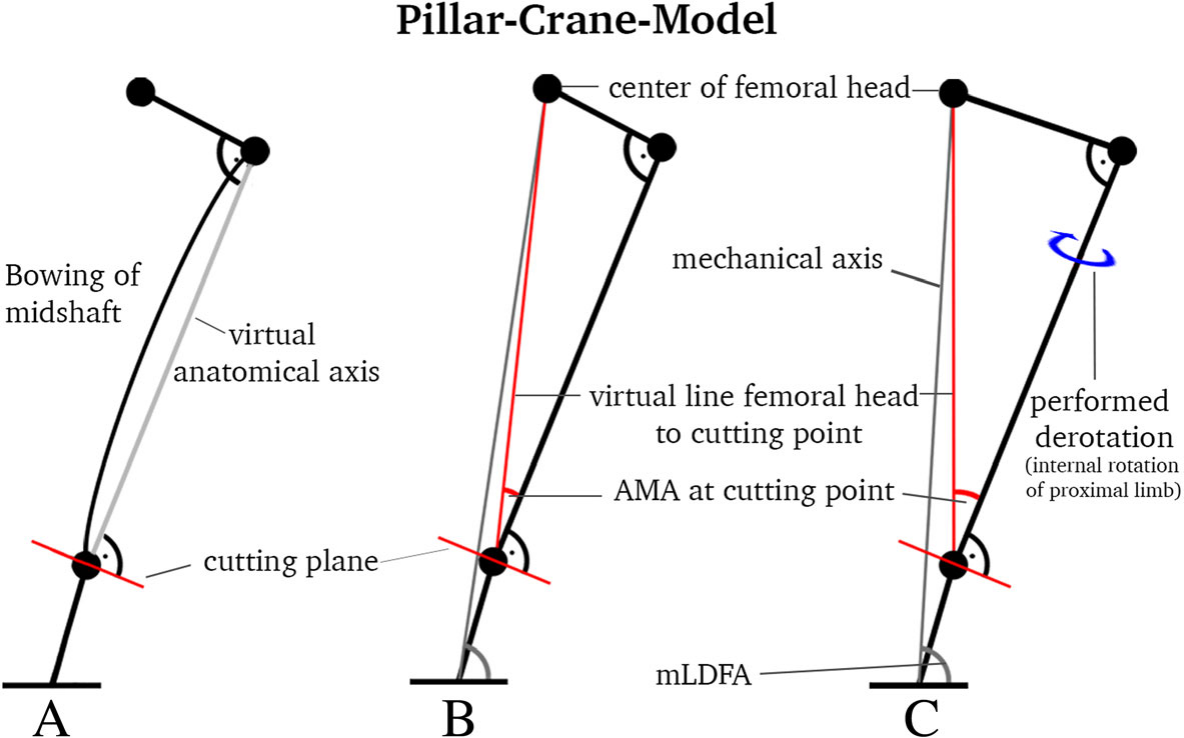
Pillar-Crane-Model frontal view; **a** virtual anatomical axis regardless of femoral bowing; **b** introducing “AMA at cutting”, which is needed for exact calculations; **c** when derotation of the proximal limb is performed, increase of AMA at cutting, and increase of mLDFA will occur

The cantilever arm of a construction crane performs rotation around a stationary pillar. This model was transferred to investigate the theoretical effect of a derotational osteotomy in the frontal plane. For better understanding, the femoral condyles and knee joint remain fixed while the proximal limb (shaft, neck and femoral head) undergoes rotation. An external rotation of the distal limb, which can also be thought of as an internal rotation of the proximal limb, corrects increased antetorsion. In order to avoid any frontal or sagittal changes to the anatomical axis, the cutting plane has to be perpendicular to the proximal shaft with respect to the greater trochanter. This is the virtual anatomical axis (Fig. 1a, b). For reproducible measurements, we defined this axis by two middle points (proximal at the greater trochanter, distal at the proposed cutting plane), which can be addressed from a frontal and a sagittal view in either way. No actual lengths are needed when calculations are done with at least two known angles in a perpendicular triangle. Therefore, measurement of the femoral-neck-angle was not taken into account as it was set to 90°, and did not interfere with calculations of the anatomical mechanical axis (AMA). In order to calculate the effect on frontal mechanical axis by a performed derotation, the angle “AMA at cutting” is introduced (Fig. 1b, c), and calculations were transferred to mLDFA (mechanical lateral distal femur angle) and aLDFA (anatomical lateral distal femur angle). AMA at cutting was needed to perform exact trigonometrical calculations in a perpendicular triangle made out of the lines from the middle point of the cutting plane towards the anatomical proximal shaft and a line towards the femoral head, which is revealed by the afore-mentioned crane model. Sagittal changes to the anatomical axis were not explored with this model, as the anatomical axis would not change in theory due to a perpendicular osteotomy. Trigonometrical formulas were processed with Mathematica (Wolfram, Version 11.1, 2016), and tables were created with Excel (Microsoft, Version 15.36, 2017).

### Transfer of “AMA at cutting”

Mechanical and anatomical axes normally cross each other within the range of the condyles, as demonstrated by Paley [18]. In order to transfer the changes on AMA at cutting on to the mechanical axis (mLDFA), lengths of the femur had to be taken into account. Considering both the standard femur length (463 mm), as described by Strecker et al. [19] on over 500 femora, and the standard cutting location of distal femoral osteotomies (70 mm above the joint line), a ratio was calculated. This ratio represents the ratio of AMA at cutting to AMA, defined as the AMA ratio in a perpendicular triangle (Fig. 1c).

### Specimen preparation

**Fig. 2:**
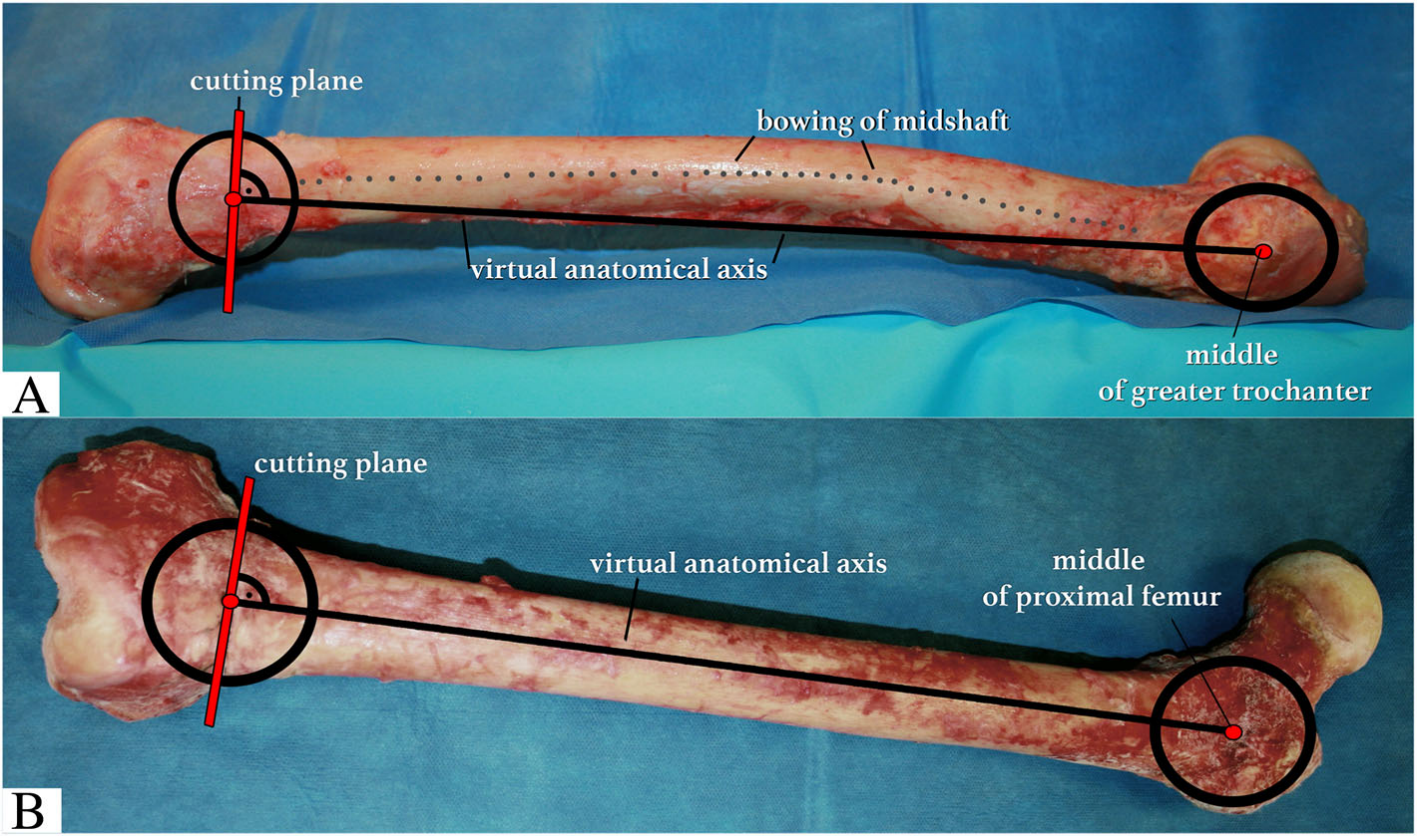
Overview of cadaveric femur shaft from frontal and lateral view for definition of the proximal middle point of the shaft and distal middle point of the shaft at the proposed cutting plane, **a** lateral view, virtual anatomical axis (black) and perpendicular cutting plane (red), despite bowing (dotted line); **b** frontal view, virtual anatomical axis (black), perpendicular cutting plane (red)

Eight different torsion angles were assessed using two human cadaver femora (left and right). Femora were stripped of skin, soft tissue and muscle. CT scan was performed and analysis of torsion was completed using the method proposed by Waidelich [20]. This analytic axial slicing technique was also utilized in several previous studies and is still commonly used [3, 21]. A single cut osteotomy was performed with an oscillating saw from lateral. The height of the cutting plane was chosen to be 70 mm from the distal joint line, as is done in clinical practice with regards to different plate designs. Angulation of the cutting plane was determined as follows (Fig. 2a): In the lateral view, a virtual line was drawn from the middle of the greater trochanter to the distal femur where it intersected with the middle of a perpendicular cutting plane. Furthermore, the cutting plane was oriented perpendicular to the anatomical axis in the frontal view (Fig. 2b).

In order to create different increased antetorsion angles, external rotation of the proximal limb by 10°, 20°, and 30° was performed and clinically observed by two k-wires and a goniometer as described by Hinterwimmer et al. [10]. Anterior-posterior radiographs of the specimens with the posterior femoral condyles resting flat on a x-ray-grit were taken with a C-arm (GE Medical Systems Inc.). These images were precisely combined generating a panoramic view of the entire femur. An open-source dicom software (OsiriX, PIXMEO SARL, Switzerland) was used for angle measurements as described by Strecker [22]: mLDFA (mechanical lateral distal femur angle), aLDFA (anatomical lateral distal femur angle), AMA (anatomical mechanical angle), and AMA at cutting (anatomical mechanical angle at cutting point). All measurements were made to the tenth of a degree. The study was reported to the institutional review board (IRB) and it was documented that no IRB approval was required (de-identified specimen do not constitute human subjects research).

### Statistical analysis

Standardized angle values of the mathematical calculation are shown in a table for meter-reading and implementation into surgical procedure. Descriptive statistics including mean and standard deviation (SD) were calculated to characterize the frontal angle measurements at different torsion angles of the specimens. As this is a preliminary concept study with different angle status on two cadavers, no inferential statistical analysis was performed.

## Results

**Fig. 3:**
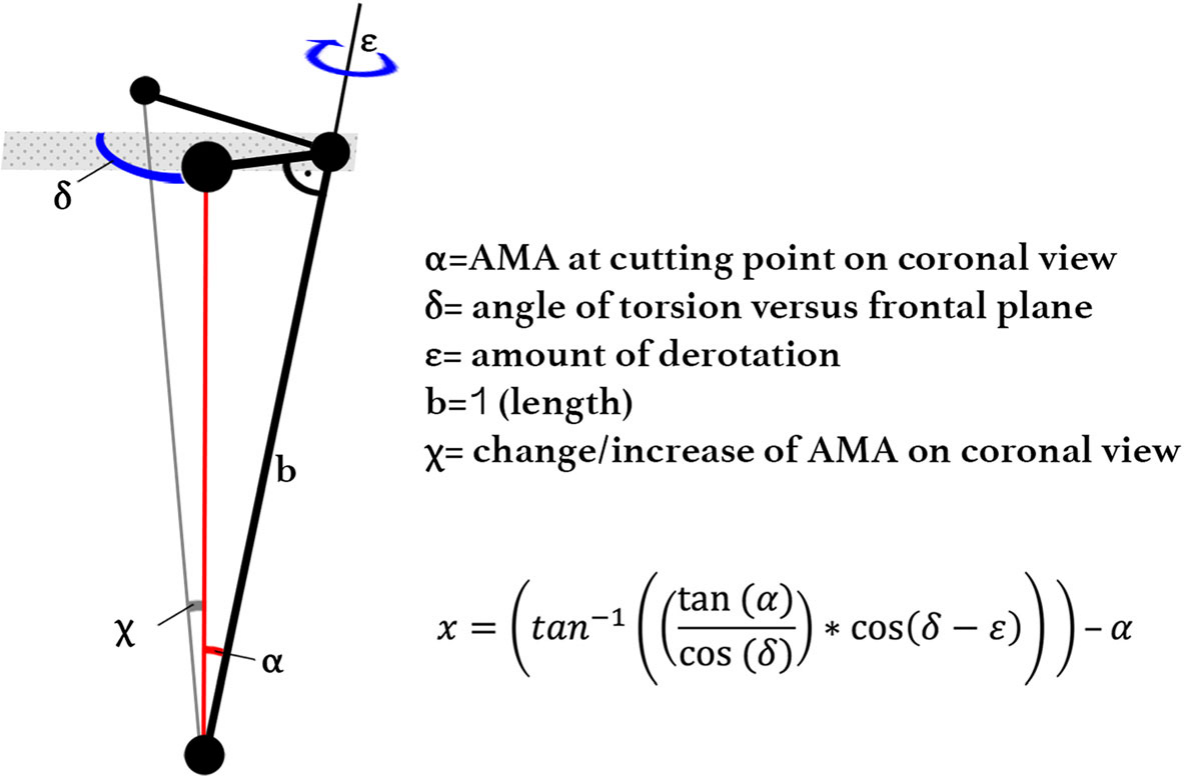
Equation of change of AMA at cutting point

Trigonometrical calculations were done to forecast the angular changes on AMA at cutting with regards to torsion and derotation (Fig. 3). Standard values of torsion angle, derotation angle, and forecast of change of AMA are shown in Table 1. This can be used as a meter-reading table in clinical practice when cutting plane is selected perpendicular to the virtual shaft axis. Specimens showed a mean increase of AMA from 4.8° to 6.3° and mLDFA from 85.2° to 86.7 after derotation by 30°. Throughout the derotation procedure, aLDFA remained at 80.4° ± 0.4°SD. After a derotation of 30° was performed from 39.7° to 9.7° torsion angle, AMA increased by +1.5°. The same increase was also observed on mLDFA (86.7° - 85.2° = 1.5°), knowing that aLDFA remains steady, because of its perpendicular rotation axis (Table 2).

**Table 1:** The AMA table; standardized values: Measured antetorsion (top), planned derotation (top), and measured AMA on a frontal plane view (left), equal a defined increase of AMA, which leads to the same increase of mLDFA in an optimal perpendicular cut

Antentorsion (°)	25	30	30	35	35	40	40	40	45	45	45
Derotation (°)	10	10	15	15	20	20	25	30	20	25	30
AMA (°)											
3	0.2	0.3	0.3	0.4	0.5	0.7	0.8	0.9	0.8	1.0	1.1
3.5			0.4	0.5	0.6	0.8	0.9	1.0	1.0	1.1	1.3
4	0.3	0.3	0.5	0.6	0.7	0.9	1.0	1.1	1.1	1.3	1.5
4.5			0.5	0.7	0.8	1.0	1.2	1.3	1.3	1.5	1.6
5	0.3	0.4	0.6	0.7	0.9	1.1	1.3	1.4	1.4	1.6	1.8
5.5			0.6	0.8	1.0	1.2	1.4	1.6	1.5	1.8	2.0
6	0.4	0.5	0.7	0.9	1.1	1.3	1.5	1.7	1.7	2.0	2.2
6.5			0.7	0.9	1.2	1.5	1.7	1.8	1.8	2.1	2.3
7	0.5	0.6	0.8	1.0	1.2	1.6	1.8	2.0	1.9	2.3	2.5
7.5	0.5	0.6	0.9	1.1	1.3	1.7	1.9	2.1	2.1	2.4	2.7
The AMA Table	Change of AMA (°) = varus increase

**Table 2:** Average measurements of specimens at four different torsion angles, all values in degrees (°)

Torsion	mLDFA	aLDFA	AMA	AMA at cutting
39.7	85.2	80.4	4.8	5.7
29.7	85.8	80.4	5.5	6.7
19.7	86.6	80.6	6.0	7.1
9.7	86.7	80.4	6.3	7.4

**Fig. 4:**
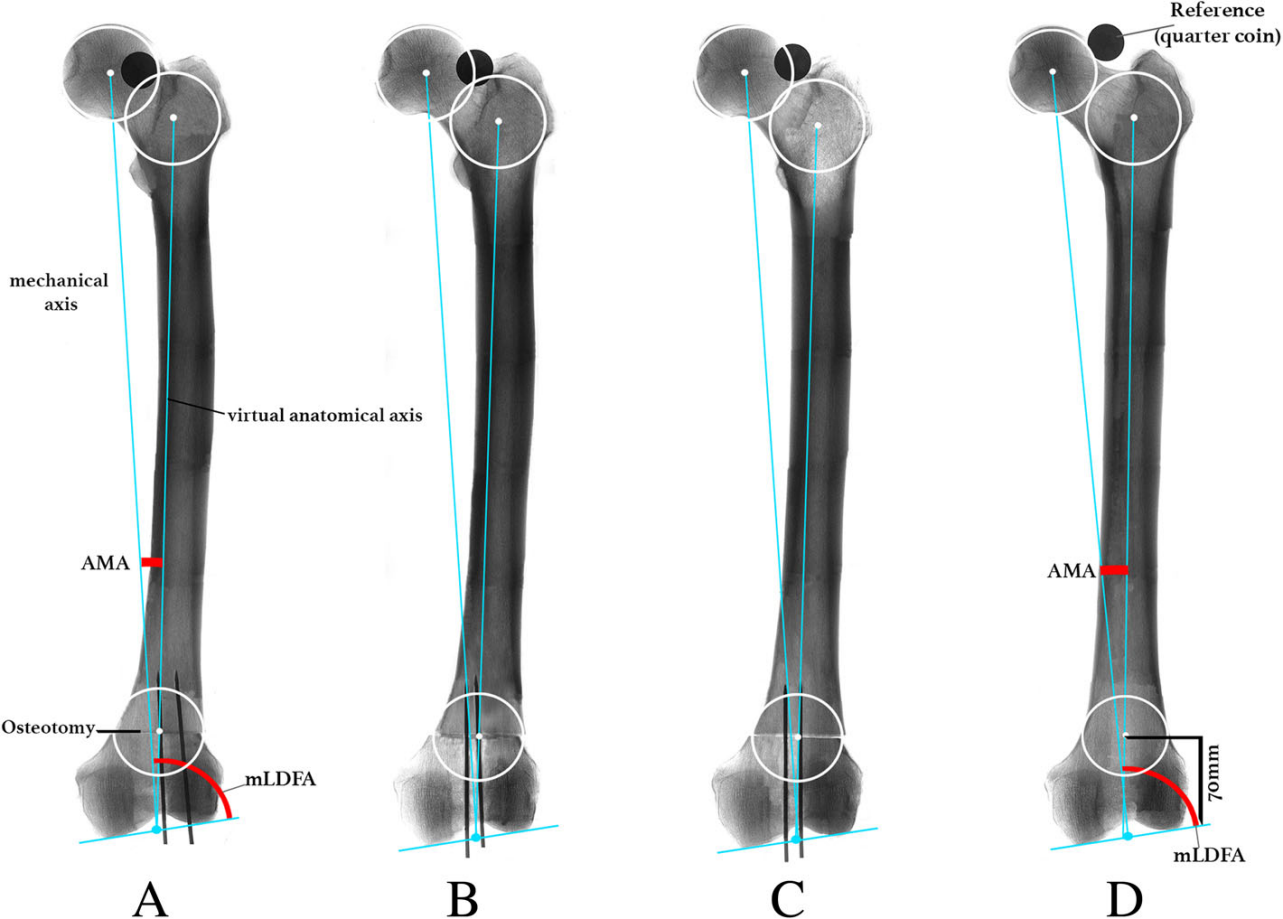
frontal view x-ray; four different torsion angles on one specimen. **a** torsion = 37°, AMA = 4.9°, mLDFA = 84.6°; **b** torsion = 27°, AMA = 5.4°, mLDFA = 85.7°; **c** torsion = 17°, AMA = 5.8°, mLDFA = 86.1°; **d** torsion = 7°, AMA = 6.4, mLDFA = 86.4°

A frontal view x-ray of the left femur at four different torsion angles is shown in Fig. 4. Regardless of bowing of the femur, the virtual anatomical axis remains the same in an optimal perpendicular cut. The calculated ratio of AMA at cutting to AMA for standard femur lengths equals: (463 mm – 70 mm)/463 mm = 0.85. With regards to our specimen observations as a control, this AMA ratio at eight different torsion angles showed a comparable mean value of 0.84 (SD ± 0.02).

## Discussion

The most important findings of the present study were that a correct reference of the cutting plane regarding the virtual anatomical shaft can avoid unintended valgus malalignment in the frontal plane in derotational osteotomies of the distal femur. This study used a novel model, called the Pillar-Crane-Model, and showed proof by trigonometrical calculations and exemplarily with the use of femur cadavers.

Adjusting the correct cutting plane in vivo is challenging in derotational osteotomy at the femur. Several studies describe their surgical technique on how to perform a distal femur derotational osteotomy [3, 4, 7, 10, 21]. But exact guidance on frontal and sagittal view of their performed cut cannot be found. Theoretically, an osteotomy perpendicular to the mechanical axis on both planes will not affect alignment on frontal and sagittal axis. The mechanical axis can be verified on a frontal view x-ray during surgery. However, the sagittal view does not reflect the mechanical axis, and reference for the cut from a lateral approach, as is usually done in clinics, cannot be reproducibly performed. Furthermore, a recent study from Nelitz et al. showed in a computed model that increased valgus malalignment can occur in distal femoral derotational osteotomies [11]. On a lateral view, the anatomic shaft axis can differ proximally versus distally by 7° or more because of midshaft bowing, which is called antecurvation. In these cases, if angulation of the cutting plane is perpendicular to the distal shaft axis, a derotation of the femur will lead to an increased valgus malalignment, the severity of which depends on the amount of derotation. As we present in our concept study, correct angulation of the cutting plane perpendicular to the virtual anatomical axis is key to avoiding an unintended result.

Lee et al. showed in several computer simulations that unexpected angular or rotational deformity can occur during rotational osteotomies [16]. They stated that femoral antecurvation and femoral bowing can affect the alignment of the lower leg and that osteotomy on the anatomical shaft, proximal or distal, influences mechanical axis. Two dimensional radiographs combined with a surgical approach from one side make it complex to intraoperatively predict three-dimensional effects due to rotational osteotomies.

The current study helps to explain why reference of the osteotomy plane to the shaft is the most important step in avoidance of unintended changes on axes. The Pillar-Crane-Model, which is a perpendicular cutting line to the virtual anatomic shaft axis will lead to a slight increase of AMA and will not aggravate a valgus malalignment. For exact measurements, AMA at cutting would be the desired angle. But for clinical practice a simplified table for normal AMA can be used, although mechanical and anatomical axes do not always cross at the joint line. Furthermore, basic drawings of the Pillar-Crane-Model show that midshaft bowing can be neglected when the cutting plane is chosen perpendicular to the virtual anatomical shaft. Our specimen model showed a standard deviation of 0.4° regarding eight measurements of aLDFA. When derotation is performed by up to 20°, a clinically insignificant increase in varus occurs. However, increase of valgus malalignment can be avoided with this model.

This method demonstrates the importance of an exact radiograph of the knee joint in the frontal plane in order to understand changes on coronal alignment when rotational osteotomies are performed. It is likely that these factors can differ in a clinical setup and lead to a certain margin of error of the angle measurement. Methods for torsion measurement are well investigated and show reliable results in terms of intra- and inter-observer agreement as Kaiser et al. described; however, depending on the measurement technique, different threshold values should be considered in clinical use [23]. Waidelich et al. illustrated a technique for torsion measurement which is commonly used: [3, 20–22]. On the proximal side, a line is drawn through the center of the femoral head on one slide and through the middle of the greater trochanter on a second slide. This suits our model regarding the virtual anatomical axis, which is a line through the middle of the femur shaft and greater trochanteric complex.

There are several limitations of this concept study. We provide a simplified mathematical approach and table for meter-reading to a complex intraoperative problem. The anatomy in patellofemoral malalignment can be highly variable. Hence, the clinical relevance is not given yet. Our exemplary study on two femoral cadavers help to illustrate the model, but does not allow to generalize the findings, yet. Because of the small sample size and measurement error no statistical relevant data is shown. The biomechanical nature of the study contains a perfect overview of the anatomy from the frontal and lateral view in order to navigate the perfect cutting angle. Further studies have to show the practicability of the reference technique. In surgery, the greater trochanter as a proximal reference can be found by palpitation when the patient is lying in a supine position. Additional, a tensioned suture or an alignment rod could be used to asses this line from proximal to distal. Further evaluation of cadavers with soft tissue might help to investigate its feasibility and accuracy in a clinical setup. Another limitation lies in the before mentioned simplification of the AMA at cutting versus AMA. For clinical relevance, transfer of AMA at cutting onto mLDFA is key to an easy understandable way. Sagittal changes on axis were not investigated with this model. However, regarding a perpendicular cutting line to the virtual anatomical axis, no change (extension or flexion) is supposed to occur when the anatomical line drawn from the greater trochanter to the distal shaft. Furthermore, the results suggest the need for an additional study with simulation on a larger sample size and transfer of the model onto pathological femur anatomy.

## Conclusions

With the use of this model for surgical guidance and anatomic reference, unintended changes on frontal malalignment can be avoided. When the cutting plane is considered to be perpendicular to the virtual anatomical shaft from a frontal and lateral view, a slight increase on mLDFA (increased varus) results when a derotational osteotomy of the distal femur is performed.

## Abbreviations

aLDFA: Anatomical lateral distal femur angle
AMA: Anatomical mechanical axis
mLDFA: Mechanical lateral distal femur angle
